# Safety and performance of MR-conditional pacing systems with automated MRI mode at 1.5 and 3 Tesla

**DOI:** 10.1007/s00330-023-09650-9

**Published:** 2023-05-17

**Authors:** Jean-Nicolas Dacher, Patrick Langguth, David Adam, Walther-Benedikt Winkler, Julio Martí-Almor, Günther Prenner, María Emilce Trucco, Amir Kol, Meixiang Xiang, Donato Melissano, Hanan Fawaz, Dennis H. Lau

**Affiliations:** 1 Department of Radiology, Normandie Univ, UNIROUEN INSERM U1096 and CHU Rouen, 37 Boulevard Gambetta, 76000 Rouen, France; 2 Department of Radiology and Neuroradiology, University Hospital Schleswig-Holstein, Campus Kiel, Kiel, Germany; 3 Monash Cardiac Rhythm Management Department, Monash Heart, Monash Medical Centre, Melbourne, Australia; 4 Medical Department II With Cardiology and Intensive Care Medicine, Klinik Landstrasse, Vienna, Austria; 5 Department of Cardiology, Hospital del Mar (IMAS-UAB), Barcelona, Spain; 6 Universitätsklinik Für Innere Medizin, Klinische Abteilung Für Kardiologie, Medizinische Universität Graz, Graz, Austria; 7 Arrhythmia Section, Cardiology Department, Hospital Universitari Doctor Josep Trueta, Girona, Spain; 8 Department of Cardiology, San Camillo De Lellis Hospital, ASL Rieti, Rieti, Italy; 9 Department of Cardiology, The Second Affiliated Hospital, Zhejiang University School of Medicine, Hangzhou, China; 10 Francesco Ferrari Hospital, Casarano, Italy; 11 Clinical Affairs, MicroPort CRM, Clamart, France; 12 Centre for Heart Rhythm Disorders, University of Adelaide and Royal Adelaide Hospital, Adelaide, SA Australia

**Keywords:** Cardiac pacemaker, Artificial, Magnetic resonance imaging, Magnetic fields

## Abstract

**Objectives:**

To evaluate at 1.5 and 3 T MRI the safety and performance of trademarked ENO^®^, TEO^®^, or OTO^®^ pacing systems with automated MRI Mode and the image quality of non-enhanced MR examinations.

**Methods:**

A total of 267 implanted patients underwent MRI examination (brain, cardiac, shoulder, cervical spine) at 1.5 (*n* = 126) or 3 T (*n* = 141). MRI-related device complications, lead electrical performances stability at 1-month post-MRI, proper functioning of the automated MRI mode and image quality were evaluated.

**Results:**

Freedom from MRI-related complications at 1 month post-MRI was 100% in both 1.5 and 3 T arms (both *p* < 0.0001). The stability of pacing capture threshold was respectively at 1.5 and 3 T (atrial:: 98.9% (*p* = 0.001) and 100% (*p* < 0.0001); ventricular: both 100% (*p* < 0001)). The stability of sensing was respectively at 1.5 and 3 T (atrial: 100% (*p* = 0.0001) and 96.9% (*p* = 0.01); ventricular: 100% (*p* < 0.0001) and 99.1% (*p* = 0.0001)). All devices switched automatically to the programmed asynchronous mode in the MRI environment and to initially programmed mode after the MRI exam. While all MR examinations were assessed as interpretable, artifacts deteriorated a subset of examinations including mostly cardiac and shoulder ones.

**Conclusion:**

This study demonstrates the safety and electrical stability of ENO^®^, TEO^®^, or OTO^®^ pacing systems at 1 month post-MRI at 1.5 and 3 T. Even if artifacts were noticed in a subset of examinations, overall interpretability was preserved.

**Clinical relevance statement:**

ENO^®^, TEO^®^, and OTO^®^ pacing systems switch to MR-mode when detecting magnetic field and switch back on conventional mode after MRI. Their safety and electrical stability at 1 month post MRI were shown at 1.5 and 3 T. Overall interpretability was preserved.

**Key Points:**

• *Patients implanted with an MRI conditional cardiac pacemaker can be safely scanned under 1.5 or 3 Tesla MRI with preserved interpretability.*

• *Electrical parameters of the MRI conditional pacing system remain stable after a 1.5 or 3 Tesla MRI scan.*

• *The automated MRI mode enabled the automatic switch to asynchronous mode in the MRI environment and to initial settings after the MRI scan in all patients.*

## Introduction

Patients with cardiac implantable electronic devices (CIED) have been long contraindicated to MRI because of concerns about the potentially harmful interference between the static, gradient, and radiofrequency fields and the CIED function [1].

There is a significant need for MRI examinations in pacemaker patients, with 28.5% of patients receiving at least 1 MRI examination within 4 years post-implantation [2]. In response to this medical need, MRI-conditional pacemaker systems, including the device, leads and programming, were developed to minimize MRI-related risks. These pacemakers were designed with no known hazards in the MRI environment within the specified conditions of use.

A number of studies [2–8] have evaluated and confirmed the safety and performance of MRI at 1.5 T in patients with MRI conditional pacing systems. However, scientific evidence is weaker for higher magnetic fields. A few small, prospective, observational studies [9–11] (≤ 20 patients) implanted with an MRI conditional pacing system reported no device-related safety issues at 2 or 3 T. However, robust clinical data concerning the safety and performance of MRI at a higher magnetic field in patients with MRI conditional pacing systems is lacking.

To evaluate the clinical safety and performance of the ENO^®^, TEO^®^, or OTO^®^ MRI conditional pacing system (MicroPort CRM^®^) at 1.5 and 3 T, an interventional, prospective, multicenter, two-arm, parallel study was conducted. This study investigated the freedom from MRI-related complications and the stability of the lead electrical performances at 1 month following the MRI scan. In addition, functioning of the automated MRI mode (Auto MM) and image quality (IQ) were evaluated.

## Materials and methods

### Study design and population

The CAPRI (clinical safety of the ENO^®^, TEO^®^, or OTO^®^ pacing system when used under 1.5 and 3 T specific MRI conditions without scan exclusion zone) study (ClinicalTrials.gov Identifier: NCT03811691; EUDAMED: CIV-19–04-027,946) was an interventional, prospective, multicenter, open-label, two-arm parallel non-comparative non-randomized study conducted in Europe and Asia–Pacific to confirm the clinical safety and performance of the ENO^®^, TEO^®^, or OTO^®^ MRI conditional single chamber rate response (SR) or dual chamber rate response (DR) pacemakers with VEGA^®^ pacing leads (Model R45, R52 or R58) in the MRI environment. The study took place between July 2019 and October 2021.

MRI conditional devices were implanted according to current applicable guidelines [12, 13]. The implanted pacing system had to meet the following requirements at the inclusion visit: battery impedance < 5 kΩ, pacing capture threshold (PCT) ≤ 2 V at 0.35 ms, lead impedance 200–3000 Ω, no diaphragmatic or pectoral stimulation at 5 V/1 ms, P-wave minimum sensed amplitude ≥ 1 mV for subjects with sinus rhythm, R-wave minimum sensed amplitude ≥ 4 mV for subjects with spontaneous conduction rhythm. Exclusion criteria were as follows: (1) other active or abandoned cardiac implants; (2) other active or passive non-MR conditional devices implanted such as metallic foreign body; (3) history of brain aneurysm with ferromagnetic clipping; (4) cochlear implants; (5) tattoos located in the body area where coil is placed; (6) cardiac surgery or medical MRI examination planned within the 3 months of inclusion; (7) age < 18 years; (8) known pregnancy, women breastfeeding or in childbearing age without an adequate contraceptive method.

The study was approved by the institutional review board/ethics committees at each center and conducted in accordance with the Declaration of Helsinki and Good Clinical Practice (ISO 14155:2011). All patients gave written informed consent.

### MRI procedure

MRI scan was performed for the purpose of the study without injection or sedation. In preparation of the MRI scan, the MRI mode of pacemaker was activated and set to “auto” by the cardiologist, allowing automatic switch to programmed asynchronous pacing mode when the magnetic field of the MRI scanner is detected and back to initial configuration when the patient gets out of the magnetic field. Patients eligible at the inclusion visit underwent an MRI scan at either 1.5 or 3 T with one of the 4 commercially available scanner brands manufactured by Siemens^®^, General Electric^®^, Philips^®^, or Canon^®^ configured with a horizontal cylindrical bore magnet with static magnetic field strength of 1.5 T or 3 T (excitation radiofrequency close to 64 MHz or 128 MHz). Maximum gradient slew rate of 200 T/m/s per axis, gradient amplitude of 50 mT/m per axis and spatial gradient of the static magnetic field of 20 T/m were allowed. For a static magnetic field of 3 T, only whole body transmit coil operating on circularly polarized radio frequency (RF) excitation was authorized. Whole body averaged specific absorption rate (SAR) was limited to 2.0 W/kg (3.2 W/kg for head scanning) and the maximum duration of radio frequency exposure was 40 min. Receive-only local coils were used. During MRI scanning, an external defibrillator was at hand in case of an emergency and patients were continuously monitored.

Based on radiology equipment, sites were preassigned to perform either a 1.5 or 3 T MRI scan. In agreement with the patient, the type of MRI examination (brain, cervical spine, shoulder, or cardiac MRI) was selected from a list of protocols predefined by the investigator. Pulse sequences were designed for their clinical relevance in accordance with routine imaging protocols with a cumulative MRI scan duration of circa 30 min (gradient and radio frequency field exposure) and at least 1 scan with near-maximal SAR value.

Procedure was not repeated in case of non-interpretability or if the examination was interrupted for any reason. SAR values were collected during the scan. The site radiologist reviewed independently MR images.

### Study objectives and endpoints

#### Primary endpoint

The primary endpoint assessed the freedom from MRI-related complications at 1 month post-MRI scan defined as any serious adverse event (SAE) that led to death or to an invasive intervention, or any device deficiency (DD) occurring during and/or after the MRI scan that caused the termination of any significant device function (e.g., loss of capture/sensing, lead damage, pacemaker damage leading to irreversible malfunction or replacement procedure). All SAEs and DDs were adjudicated by a Clinical Event Committee (CEC) and classified as being either complications or observations and according to their relationship to the pacing system (pacemaker and/or lead) hardware and software, and/or to the MRI procedure. Only complications classified as being probably related, definitely related or relationship unknown to both the MRI procedure and the pacing system were considered.

#### Secondary endpoints

Four secondary study endpoints between pre-MRI and 1 month post-MRI were used including (1) ventricular and (2) atrial PCT stability at 0.5 ms (defined as ≤ 0.5 V at 0.5 ms), (3) ventricular and (4) atrial sensed amplitude stability (defined as ≤ 50% decrease).

#### Other endpoints

##### Lead electrical performance

Atrial and ventricular PCT at 0.5 ms, minimum sensed amplitude and impedance were assessed before and shortly after the MRI scan and at 1-month follow-up.

##### MRI-related adverse events (AEs)

Additional safety data were all AEs collected at MRI visit, classified as observations and adjudicated by the CEC as being probably/definitely related to MRI.

##### Proper functioning of the automated MRI mode

Auto MM was assessed by the pacing system’s capability of automatically switching (1) to programmed asynchronous mode when detecting the external magnetic field, and (2) to initial settings after termination of the scanning. Switch to asynchronous mode and to initial settings was confirmed by device file analysis. Data was classified as evaluable when the MRI mode was programmed to “auto” before the MRI scan with a pacing rate higher (+ 10 bpm) than subject’s spontaneous pacing rate and the device file including the Holter information was available.

##### Interpretability of the MRI scan images

Image quality (IQ) was assessed onsite by the certified radiologist in charge of the MR exam by means of the following 5-level Likert scale: (1) excellent; (2) good, insignificant device artifacts; (3) medium, significant device artifacts not limiting the interpretation; (4) poor; significant device artifacts limiting the interpretation; (5) image not interpretable.

### Sample size and statistical analysis

To provide a statistical power of at least 80% for the primary safety endpoint, a sample size of 114 patients was required for each study arm (1.5 or 3 T) to reject the null hypothesis of insufficient safety of the device, defined as the proportion of patients free from MRI-related-complications at 1 month post-MRI ≤ 90%, assuming an expected performance of 97%, and using a 0.025 one-sided alpha. With an expected dropout rate of 15%, the number of patients to be included was therefore 135 for each study arm.

The primary safety and secondary performance endpoints were evaluated in the Full Analysis Set (FAS) population, i.e., patients of the included population who underwent MRI examination, partially or totally, and with evaluable data at 1-month post-MRI visit. For the primary safety endpoint, the null hypothesis of insufficient safety of the device, defined as a proportion of patients free from MRI-related complications at 1 month post-MRI ≤ 90%, was tested by means of a one-sided exact binomial test at the significance level of 0.025. The endpoint was met if the lower bound of the two-sided 95% confidence interval (CI) > 90%. For the secondary performance endpoints, the null hypothesis of insufficient pacing capture threshold, or lead sensed amplitude, defined as the proportion of successful lead pacing capture threshold, or lead sensed amplitude, at 1 month post-MRI ≤ 90%, was tested by means of a one-sided exact binomial test at the significance level of 0.025. These secondary endpoints were met if the lower bound of the two-sided 95% confidence interval (CI) > 90%. Primary and secondary endpoints are presented with 95% CI.

Descriptive statistics were applied to assess all other study endpoints. Continuous quantitative parameters are presented as mean ± standard deviation (SD). Median and 1st and 3rd quartiles (Q1, Q3) are used for variables not normally distributed. Qualitative parameters are presented as number and frequency (%). Statistical analyses were performed with SAS version 9.4 or above (SAS Institute).

## Results

### Study population and MRI procedure

**Fig. 1:**
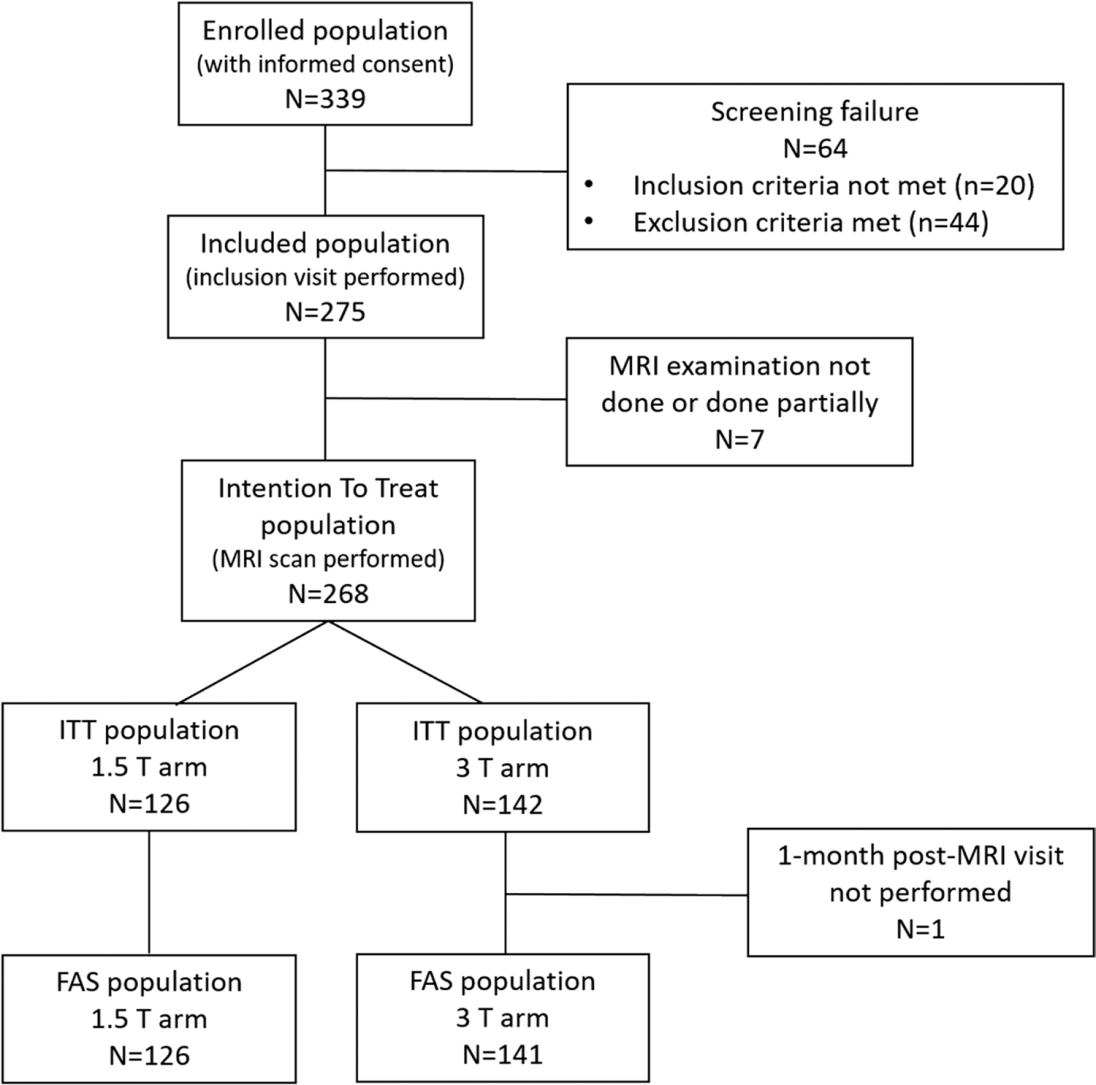
Flowchart of the patient disposition and study population. ITT, intention to treat; FAS, full analysis set

Two hundred seventy-five patients (127 at 1.5 T and 148 at 3 T) were included at 25 sites (Fig. 1). Major reason of screen failure was patient’s refusal to continue participation in the study during the COVID-19 pandemic. Of the included population, 126 patients in the 1.5 T arm and 142 in the 3 T arm underwent the MRI examination (intention to treat (ITT) population). One patient of the 3 T arm did not perform the post-MRI visit at 1 month, resulting in the inclusion of 126 and 141 patients in the full analysis set (FAS) population of the 1.5 T and 3 T arms, respectively.

Demographics of the ITT population are shown in Table 1. Population characteristics were typical of patients requiring a pacing system and comparable in the two study arms. The percentage of patients with a co-existing MR conditional device implanted such as coronary stent, hip replacement, knee prosthesis, or sternal wires was 4% in the 1.5 T arm and 7% in the 3 T arm. The proportion of dual chamber ENO^®^, TEO^®^, or OTO^®^ pacemakers was 81.7% in the 1.5 T arm and 90.8% in the 3 T arm.

**Table 1:** Baseline characteristics of the ITT population (*N* = 268) presented per study arm

Variable	1.5 Tesla MRI *N* = 126	3 Tesla MRI *N* = 142
Sex, male	79 (62.7)	90 (63.4)
Age (years)	75.5 ± 9.7	74.6 ± 10.4
BMI (kg/m)	27.4 ± 5.1	27.5 ± 5.3
History of conduction disorders		
Atrioventricular block	63 (50.0)	95 (66.9)
Sinus node dysfunction	51 (40.5)	50 (35.2)
Atrial arrhythmias	41 (32.5)	55 (38.7)
Ventricular arrhythmias	7 (5.6)	5 (3.5)
Other	14 (11.1)	22 (15.5)
Comorbidities		
Hypertension	86 (68.3)	111 (78.2)
Diabetes mellitus	23 (18.3)	47 (33.1)
Renal insufficiency	27 (21.4)	25 (17.6)
Cardiomyopathy	25 (19.8)	15 (10.6)
Stroke	14 (11.1)	6 (4.2)
Myocardial infarction	4 (3.2)	15 (10.6)
Chronic obstructive pulmonary disease	3 (2.4)	7 (4.9)
Pericarditis	0 (0.0)	1 (0.7)
Type of intervention		
Initial	121 (96.0)	139 (97.9)
Replacement	4 (3.2)	3 (2.1)
Upgrade	1 (0.8)	0 (0.0)
Pacemaker model		
DR	103 (81.7)	129 (90.8)
SR	23 (18.3)	13 (9.2)
Right atrial lead		
Yes	104 (82.5)	129 (90.8)
No	22 (17.5)	13 (9.2)
Right ventricular lead		
Yes	125 (99.2)	142 (100)
No	1 (0.8)	-
Co-existing MR conditional device implanted		
None	119 (94.4)	130 (91.5)
1	5 (4.0)	8 (5.6)
2	2 (1.6)	3 (2.1)
3	0 (0)	1 (0.7)
Type of MRI protocol performed		
Brain	58 (46.0)	75 (52.8)
Cervical spine	19 (15.1)	41 (28.9)
Cardiac region	38 (30.2)	9 (6.3)
Shoulder	11 (8.7)	17 (12.0)
MRI exam duration (min)	31.5 ± 8.0	33.0 ± 9.3
Cumulative RF exposure (min)	24.5 ± 5.8	26.6 ± 6.0
AM		
Yes No	119 (94.4) 7 (5.6)	136 (95.8) 6 (4.2)

Values are presented as mean ± SD or as *n* (%)

*BMI* body mass index; *MRI* magnetic resonance imaging; *RF* radio frequency

Data related to the MRI procedure are presented in Table 1. Scanning was prematurely terminated in 4 patients (1 at 1.5 T due to claustrophobia; 3 at 3 T due to claustrophobia or refusal to continue the scan).

### MRI-related events

All patients were free from MRI-related complications at 1 month post-MRI so the primary safety objective was met at both 1.5 and 3 T. Table 2 shows all MRI-related safety events in the ITT population of the 1.5 and 3 T study arm. All devices switched to asynchronous mode when the magnetic field was detected and resumed to normal mode after the MRI exam.

**Table 2:** MRI-related safety events in the ITT population (*N* = 268) presented per study arm

	1.5 Tesla MRI *N* = 126		3 Tesla MRI *N* = 142	
	#patients (%)	#events	#patients (%)	#events
SAE related to MRI	0 (0.0)	0	0 (0.0)	0
Non-serious AEs related to MRI procedure	3 (2.4)	3	8 (5.6)	8
Claustrophobia	1 (0.8)	1	2 (1.4)	2
Heat sensation on MRI examination area	0 (0.0)	0	2 (1.4)	2
Headache	0 (0.0)	0	1 (0.7)	1
Itching sensation on device pocket^a^	0 (0.0)	0	1 (0.7)	1
Pocket warming^b^	2 (1.6)	2	0 (0.0)	0
Subject movement during MRI scan	0 (0.0)	0	1 (0.7)	1
Vasovagal reaction	0 (0.0)	0	1 (0.7)	1

^a^Adjudicated as related to the pacing system

^b^Adjudicated as probably related to the pacing system

*MRI* magnetic resonance imaging

### Performance of the pacing system under MRI

The proportion of successful ventricular PCT stability at 1 month post-MRI was 100% in 1.5 T (111/111 patients; 95% CI: 96.7% to100%; *p* < 0001) and 3 T arms (122/122 patients; 95% CI: 97.0% to 100%; *p* < 0001). The proportion of successful atrial PCT stability at 1 month post-MRI was 98.9% (86/87; 95% CI: 93.8 to 100%; *p* = 0.001) in the 1.5 T arm and 100% (95/95; 95% CI: 96.2 to 100%; *p* < 0.0001) in the 3 T arm.

The proportion of successful ventricular sensed amplitude stability at 1 month post-MRI was 100% (106/106 patients; 95% CI: 96.6 to 100%; *p* < 0.0001) in the 1.5 T arm and 99.1% (108/109 patients; 95% CI: 95 to 100%; *p* = 0.0001) in the 3 T arm. The proportion of successful atrial sensed amplitude stability at 1-month post-MRI was 100% (87/87 patients; 95% CI: 95.8 to 100%, *p* = 0.0001) in the 1.5 T arm and 96.9% (94/97 patients; 95% CI: 91.2 to 99.4%; *p* = 0.01) in the 3 T arm.

All secondary performance objectives were met. Atrial and ventricular PCT and sensed amplitude stability was confirmed at 1.5 and 3 T.

Right atrial and ventricular lead electrical performance, i.e., PCT, sensed amplitude and impedance, at pre-MRI, immediately post-MRI and 1 month post-MRI at both 1.5 and 3 T are shown in Table 3.

**Table 3:** Lead Electrical performance parameters in the ITT population

Study arm	Electrical performance	Lead		Pre-MRI	Post-MRI	1 month post-MRI
1.5 Tesla	Pacing capture threshold (V)	RA	Mean ± SD	0.69 ± 0.20	0.69 ± 0.18	0.74 ± 0.20
		RV	Mean ± SD	0.80 ± 0.25	0.79 ± 0.25	0.82 ± 0.25
	Sensed amplitude (mV)	RA	Mean ± SD	3.69 ± 1.69	3.73 ± 1.66	3.75 ± 1.67
			Median (Q1, Q3)	3.9 (2.1, 5.1)	3.8 (2.3, 5.1)	3.8 (2.2, 5.2)
		RV	Mean ± SD	12.78 ± 2.99	12.88 ± 2.84	12.68 ± 3.10
			Median (Q1, Q3)	14.9 (10.5, 15.0)	15.0 (10.8, 15.0)	15.0 (10.5, 15.0)
	Impedance (Ω)	RA	Mean ± SD	544 ± 100	541 ± 94	550 ± 109
		RV	Mean ± SD	659 ± 170	663 ± 162	656 ± 172
3 Tesla	Pacing capture threshold (V)	RA	Mean ± SD	0.68 ± 0.20	0.68 ± 0.18	0.69 ± 0.17
		RV	Mean ± SD	0.78 ± 0.25	0.78 ± 0.23	0.80 ± 0.22
	Sensed amplitude (mV)	RA	Mean ± SD	3.77 ± 1.56	3.83 ± 1.61	3.87 ± 1.68
			Median (Q1, Q3)	3.7 (2.7, 5.1)	4.0 (2.5, 5.2)	3.7 (2.6, 5.7)
		RV	Mean ± SD	12.46 ± 3.50	12.55 ± 3.33	12.25 ± 3.51
			Median (Q1, Q3)	15.0 (10.4, 15.0)	15.0 (10.5, 15.0)	14.2 (9.6, 15.0)
	Impedance (Ω)	RA	Mean ± SD	558 ± 125	550 ± 119	558 ± 118
		RV	Mean ± SD	661 ± 181	656 ± 184	662 ± 187

*RA* right atrial; *RV* right ventricular

### Functioning of the automated MRI mode

In both 1.5 and 3 T arms, 100% of MR conditional pacing systems automatically switched to asynchronous mode when the magnetic field was detected, and reverted to initially programmed mode after the MRI exam, thus, confirming the proper functioning of the Auto MM.

### Interpretability of MR examinations

In both 1.5 and 3 T arms, Table 4 shows that 100% of MRI images were assessed as interpretable. For brain MRI, 100% and 98.6% of scan images were appraised as excellent or good under 1.5 and 3 T, respectively. For C-spine scans, 100% and 87.8% of scan images were assessed as excellent or good at 1.5 and 3 T, respectively. Image quality of cardiac examinations was good to excellent for the majority of 1.5 T MRI scans (86.8%) and medium to excellent for the majority of 3 T scans (88.8%). However, 5.3% and 11.1% of images obtained from cardiac MRI scans in the 1.5 and 3 T arm, respectively, were appraised as poor quality with significant artifacts limiting the interpretation of the examination. Twenty percent of shoulder examinations were deemed poor at 1.5 T.

**Table 4:** Interpretability of the MRI scan images presented per study arm

Image quality	1.5 Tesla MRI *N* = 125^a^				3 Tesla MRI *N* = 142			
	Cardiac *N* = 38	Shoulder *N* = 10	Brain *N* = 58	Cervical Spine N = 19	Cardiac *N* = 9	Shoulder *N* = 17	Brain *N* = 75	Cervical Spine *N* = 41
Excellent	4 (10.5)	5 (50.0)	30 (51.7)	12 (63.2)	4 (44.4)	7 (41.2)	58 (77.3)	20 (48.8)
Good	29 (76.3)	2 (20.0)	28 (48.3)	7 (36.8)	1 (11.1)	7 (41.2)	16 (21.3)	16 (39.0)
Medium	3 (7.9)	1 (10.0)	-	-	3 (33.3)	3 (17.6)	1 (1.3)	5 (12.2)
Poor	2 (5.3)	2 (20.0)	-	-	1 (11.1)	-	-	-
Not interpretable	-	-	-	-	-	-	-	-

^a^Missing data for 1 image scan of the shoulder; therefore, 125 scan images available from the ITT population

Values are presented as *n* (%)

*MRI* magnetic resonance imaging

## Discussion

This study demonstrates the safety of the ENO^®^, TEO^®^, or OTO^®^ MRI conditional pacing system in the MRI environment without scan exclusion zone. Our principal findings are as follow. First, there were no complications related to the MRI procedure and the pacing systems with no serious adverse event or device deficiency at 1 month post-MRI. Second, the pacemaker systems demonstrated excellent PCT and sensing amplitude stability at 1 month post-MRI. Third, 100% of patients switched to asynchronous mode when in the MRI environment and automatically resumed to normal mode after leaving the magnetic field. Last, 100% of MRI images were assessed as interpretable and image quality was assessed as good or excellent in > 90% of the scans. Specifically, this is the largest prospective study to date on the safety of conditional pacemaker systems at 3 T.

Patients implanted with pacemakers were previously excluded from MRI scans due to safety concerns. MRI conditional pacemakers referring to devices that pose no known hazards in a specific MRI environment under specific device and MRI scanner conditions have been developed over the last two decades [14]. Design changes included reduction of ferromagnetic content, modification of reed switches, lead redesign to reduce lead-tip heating, and implementation of circuitry filters and shielding to limit the transfer of electromagnetic effects [15]. Our results confirmed findings from previous large, prospective studies regarding the safety of MRI conditional pacemakers at 1.5 T [2–8].

Despite robust safety data on pacemaker devices at 1.5 T, the evidence for the safety of scanning patients with MRI conditional pacemakers at 3 T is limited to small series [10, 11]. The higher pulsed radio-frequency field and static magnetic field at 3 T produces increased radiofrequency-related energy deposition and higher magnetic forces, which are of particular concern in patients implanted with electronic and metallic devices. Safety concerns include tissue heating, particularly for conductive material with elongated shapes such as leads, and torque possibly leading to movement or twisting of the implant inside the patient’s body. This study is the first large prospective, multicenter trial confirming the safety of MRI conditional pacing system at 3 T. Additionally, our study showed excellent electrical performance with stability of PCT and sensing amplitude. Out of 142 patients undergoing a 3 T scan, only one patient (0.7%) experienced a non-serious event related to both the MRI procedure and pacing system, reported as itching sensation on the device pocket. This is similar to the two (1.6%) patients that reported a sensation of pocket warming in the 1.5 T arm. CAPRI study is in keeping with other studies [2, 8] with up to 3.8% non-serious observations that were possibly related to the MRI procedure (paraesthesia, palpitations, transient chest pressure, swallowing difficulty, transient changes in PCT, atrial flutter and fibrillation) at 1.5 T.

In addition to modifications in hardware, the tested trademarked MR conditional pacemaker systems are currently designed with an automated MRI mode to improve pacemaker patients care pathways. Auto MM is programmable by the specialist between 2 h and 10 days before the MR examination. Upon activation, the pacing system is set to automatically switch to programmed asynchronous pacing in the presence of a strong magnetic field and revert to initial programmed settings approximately 5 min after termination of the scan, thereby limiting grossly the time in asynchronous mode to the MRI scan duration. This is of importance as asynchronous pacing may induce life-threatening ventricular tachycardia in patients with spontaneous rhythm [16]. All devices in the CAPRI trial automatically switched to asynchronous mode when the magnetic field was detected and resumed to initial configuration after the MRI exam. No safety issues related to Auto MM were reported. In view of efficient patient care workflow, Auto MM significantly improves workflow flexibility by eliminating the need of immediate assistance of a cardiac specialist just before, during, and after the MRI scan and reducing the burden for patients and hospital staff. In addition, a recent retrospective analysis found that the Auto MM of CIED systems eliminated same-day post-MRI device interrogations in 91.4% of MRI exposures [17]. Although no strict guidelines on patient follow-up after MRI procedure are currently available, it is clear that the Auto MM improves pacemaker patients care pathways and reduces hospital resource utilization. However, it is preferable to keep the patient in a controlled medical environment until the Auto MM switches to initial configuration.

**Fig. 2:**
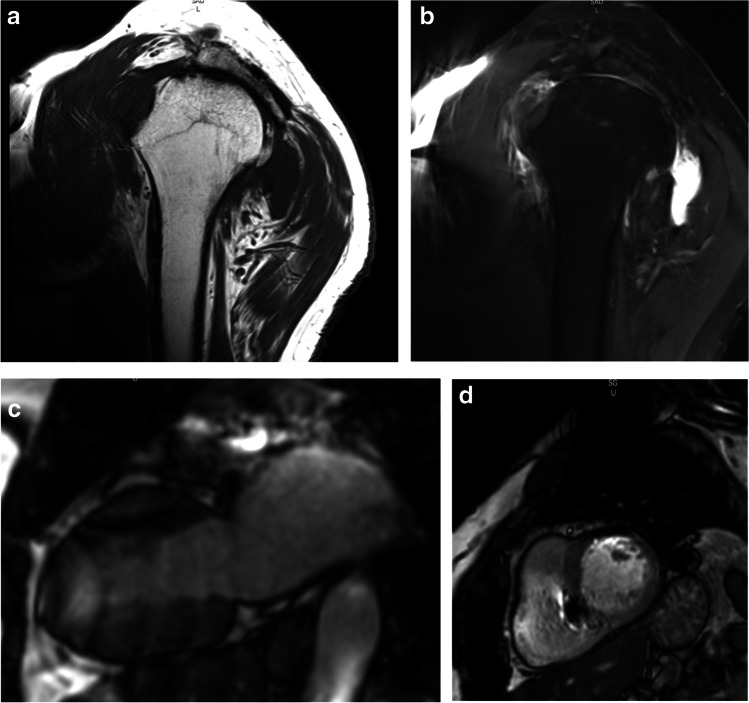
T1- (**a**) and T2-weighted (**b**) medium quality images of the left shoulder of an obese male patient performed on a 3 T Siemens equipment. Note presence of magnetic susceptibility artifacts on the left-hand side of both images. Balanced FFE (b-FFE) vertical long axis view of the heart (**c**) drawn from an examination performed in an overweight woman on a 3 T Philips equipment. Less artifacts are visible on the b-FFE short axis view (**d**) drawn from the same examination that was rated medium quality

Even if safety issues and practicability are essential, image quality remains the number one objective for radiologists. Although 100% examinations were judged interpretable in the trial (Table 4), various artifacts decreased the IQ of a subset of examinations at 1.5 and 3 T (Fig. 2 a, b, c, and d). We observed medium and poor results in 21/267 (7.8%) patients and most cases regarded either cardiac or shoulder examinations. Unexpectedly, the proportion of examinations with deleterious artifacts was not increased in patients who underwent 3 T MRIs. However, and this is one among other study limitations regarding cardiac MR, no gadolinium injection was performed in the trial though ruling out myocardial delayed enhancement is a major objective of cardiac MR examinations in patients with CEIDs [18].

It is also important to state that this study was performed under strict supervision of patients and devices and an optimal communication between cardiology and radiology teams. The results of the present study could only be extrapolated to routine practice if a strict protocol of co-operation between the radiology and cardiology departments is put in place and strictly applied at the institution level [19, 20].

## Conclusion

This large, prospective multicenter trial demonstrated the clinical safety and performance of the ENO^®^, TEO^®^, or OTO^®^ MRI conditional pacing systems at 1 month post-MRI with no scan exclusion under 1.5 and 3 T. Specifically, this study provides data on the safety and stability of the pacing system at 3 T and on the proper functioning of the automated MRI mode. In order to improve MR access of patients implanted with MRI conditional pacing systems and maximize patient safety, robust interdepartmental protocols in line with international guidelines [12] and an educated and well-trained, multidisciplinary team of radiologists, cardiologists, device applications specialists, nurses, and MRI technicians are required. Increased access of pacemaker patients to this established medical imaging technique will allow timely diagnosis of diseases and improve follow-up and therapeutic management in this vulnerable population.

## Declarations

### Guarantor

The scientific guarantor of this publication is Pr Jean-Nicolas Dacher.

### Statistics and biometry

Yann Michel, MSc, kindly provided statistical advice for this manuscript.

### Methodology

• prospective.

• observational.

• multicenter.
